# Molecular materials – towards quantum properties

**DOI:** 10.3762/bjnano.6.153

**Published:** 2015-07-08

**Authors:** Mario Ruben

**Affiliations:** 1 Institute of Nanotechnology, Karlsruhe Institute of Technology, D-76344 Eggenstein-Leopoldshafen, Germany; 2 Institut de Physique et Chimie des Matériaux, Université de Strasbourg, 23 Rue du Loess, F-67200 Strasbourg, France

Molecular materials are promising candidates for the expression of quantum properties, mainly due to the inherent monodispersity of their building blocks and the unique possibility to tailor the local environments of (natural) atoms.

Both artificial as well as natural atoms have been proposed as quantum objects in the realisation of quantum information schemes. As a result of their confinement, electrons in both natural atoms and in quantum dots, so-called artificial atoms, are characterized by the formation of discrete energy levels. Similarly, in the case of a Josephson junction, Cooper pairs are confined in the potential well of the Josephson coupling energy leading to a discrete distribution of energy levels. Hence, the junction can be considered as a superconducting artificial atom. With regard to magnetism, single spins of impurities in semiconductors as well as molecular nanomagnets have been proposed as solid-state candidates for quantum bits (qubits). The used quantum correlations (e.g., entanglement, coherence) are usually observed only on the nanometer scale and have long been recognized as an information resource for quantum communication and processing. In particular, there is a considerable motivation to produce quantum computers, and a great deal of interest from scientists working in materials science, chemistry, physics, and nanofabrication technologies has been attracted. For example, the company D-wave has demonstrated a quantum annealer that performs certain calculations sufficiently rapidly to have a consortium, which involves Google and NASA, investing staggering sums of money in one such device [1].

The main advantage of the use of the molecular approach here is that molecules are quantum objects that can be produced by synthetic tools in a large number of atomically precise copies – a requirement for the scalable exploitation of the quantum properties. Devices based on single, or small numbers of, molecules, could speed up information treatment or allow for processing schemes that have not been possible to date. In a series of recent publications, it could be shown that magnetic molecules, in particular lanthanide complexes can be considered to be spin-qubits [2] or spin qugates [3]. Moreover, it was shown that molecule-based nuclear spins are extremely well insulated from environmental perturbations, rendering them less prone to decoherence. By means of synthetic engineering the central fine-tuning of the delicate trade-off between decoupling of the quantum object for low decoherence and connecting it for the electrical read-out could be achieved [2].

Quantum computing, the manipulation of data encoded in qubits instead of bits of information such as spin states of electrons or of an atomic nucleus, has been a long standing goal of scientists. In principle, if the qubits can maintain their coherence, without being perturbed by the noise of the surrounding environments (e.g., neighbouring atoms), quantum computers could be powered to find the best solutions far more quickly than current conventional computers. In some of the proposed device materials, quantum information is carried by the spin and the orbital degree of freedom of the electron. The challenge consists in circumventing the strong coupling of the latter one with the environment in order to achieve the needed long coherence times to carry out quantum operations.

In this context, molecular quantum materials offer the additional advantage that the active quantum processing elements comprise an atomic core of one to a few open-spin ions surrounded by a shell of organic material. At low temperatures, the behavior of such molecular spin objects can be well described by simple few-level systems. Moreover, their spin degree of freedom can be sufficiently decoupled from environmental perturbations to attain long coherence times, thus making them the ideal candidates for the implementation of qubits. The organic shell can be used to fine-tune the coupling between molecular qubits as required by scalability for logical quantum gate operations.

We have gathered in the Thematic Series contributions dealing with the magnetic properties of molecules, partially under use of lanthanide metal ions und their coordination under surface confinement. By the same token, the needed surface attachment and positioning of molecular materials by self-assembly techniques is addressed – an important prerequisite for the electrical addressing of molecules by lateral graphene electrodes or vertical scanning tunnelling microscopy set-ups or e-beam lithographed gold or Prussian blue nano-arrays. Complementary, quantum chemical calculations have addressed lanthanide complexes and metal-organic frameworks.

This Thematic Series is part of a subsession of the same title, which took place at the E-MRS spring meeting in May 2014 in Lille, France. I would like to kindly thank Prof. G. Aromi (Barcelona) and Prof. A. Bousseksou (Toulouse) for co-chairing this event.

Mario Ruben

Karlsruhe, June 2015
